# Solubility of lumiracoxib in supercritical carbon dioxide

**DOI:** 10.1038/s41598-024-63416-x

**Published:** 2024-06-10

**Authors:** Firas H. Albadran, Nabeel K. Abbood, Mohammad A. Al-Mayyahi, Seyednooroldin Hosseini, Mohammed S. Abed

**Affiliations:** 1 Basrah University for Oil and Gas, Basrah, Iraq; 2 EOR Research Center, Department of Petroleum Engineering, Omidiyeh Branch, Islamic Azad University, Post Box 164, Omidiyeh, 63731-93719 Iran; 3 Chemical Engineering Department, University of Al-Amareh, Missan, Iraq

**Keywords:** Solubility, Supercritical carbon dioxide, Nimesulide, Lumiracoxib, Crossover pressure, Chemistry, Engineering, Materials science

## Abstract

This study aims to use a static-based solubility method for measuring the solubility of lumiracoxib at a temperature of 308–338 K and pressure of 120–400 bar for the first time. The obtained solubility data for lumiracoxib is between 4.74 × 10^−5^ and 3.46 × 10^−4^ (mole fraction) for the studied ranges of pressure and temperature. The solubility values reveal that the lumiracoxib experiences a crossover pressure of about 160 bar. Moreover, the measured solubility data of these two drugs are correlated with density-based semi-empirical correlations namely Bartle et al., Mendez-Santiago-Teja, Kumar and Johnstone, Chrastil and modified Chrastil models with an average absolute relative deviation of 10.7%, 9.5%, 9.8%, 7.8%, and 8.7% respectively for lumiracoxib. According to these findings, it is obvious that all of the examined models are rather accurate and there is no superiority between these models for both examined drugs although the Chrastil model is slightly better in the overall view.

## Introduction

It is well established that it is possible to increase the rate of dissolution for poorly soluble drugs by reducing the particle size (according to the dissolution equation). In other words, based on the dissolution equation derived from the film theory, the surface area, concentration gradient across the diffusion layer and the solubility of a compound are effective parameters on the dissolution rate. In general, micronizing the drug particles enhances the dissolution rate since the available surface area to the dissolving medium increases. Respecting this fact, extensive researches were performed on the micronization methods to find suitable methods without manipulating the physiochemical properties of the drug particles^1–7^. Among the possible micronization methods, supercritical fluid technologies (SFT) especially those that use supercritical carbon dioxide (SC-CO_2_) as the main solvent gain increasing attention for producing nano and micron-size particles with controlled morphology^8^.

The SC-CO_2_-based technologies are desired due to their undeniable advantages, such as being environmentally friendly, being nontoxic and producing low toxic wastes, low impact on the particle structure, high final quality, etc. In light of these unique advantages, supercritical fluid-based technologies are on the main radar of scientists for different applications of recycling valuable components from waste materials and streams such as batteries^9,10^, extraction of oil seeds such as portulaca oleracea and dracocephalum kotschyi^11,12^, measuring the solubility of drugs^13–16^, and producing nanoparticle^13,17,18^.

In the shadow of unique features, using SC-CO2 for micronization of drug particles in particular using SC-CO_2_ as the main solvent gained the attention of scientists to fabricate the nano and micron size particles using RESS which is rapid expansion of supercritical solution^17–23^, gas anti-solvent (GAS) which is gas anti-solvent, supercritical anti-solvent (SAS), etc^24–29^.

For more information and clarification, Sodeifian and Usefi^13^ provided a valuable review regarding the application of SC-CO_2_ for measuring the solubility of drugs, extracting the essential oil and seed oils besides the fabrication of nanoparticles using SC- CO_2_ as solvent and anti-solvent.

Although these methods are of great interest, one of the main common characteristics that dictate the successfulness of these methods is the substance solubility in SCF which in the case of pharmaceutical compounds, the desired fluid is carbon dioxide (CO_2_). So, it is highly important to determine the solubility of the model drug at different pressures and temperatures to conclude if the proposed micronization methods would be successful or not. On the other side, knowing the solubility of the compound in the SC-CO_2_ directly affects the size of the equipment which is highly required for economic issues. Respecting this importance, the solubility of severe al drugs and pharmaceuticals was measured at different temperatures and pressures with other researchers^18,30–49^.

According to the measured and published solubility data, one can observe that concomitant with experimental solubility measurement, it is highly beneficial if one can use the modeling approaches to correlate/interpolate/extrapolate the solubility data. In this way, different approaches such as using equations of state (EoSs)^50–54^, artificial neural network-based methods^55–57^, and semi-empirical density-based correlations are proposed and used^58–63^.

Among these possible modeling approaches, EoSs suffer some critical shortcomings if they are used for solubility prediction and modeling purposes. In detail, these models are highly dependent on the parameters including sublimation pressure, critical pressure and, temperature of the solute which their experimental values are not available. So, the users must use the predicted and estimated values of these critical parameters to introduce some intrinsic deviation into the accuracy of the predicted solubility data. As a way to eliminate these shortcomings, researchers are widely concentrated on the application of new types of predictive approaches usually called semi-empirical density-based correlations^58–63^.

These methods are highly applicable since they use thermodynamic parameters (pressure and temperature) and solution density at the specific pressure and temperature which one can measure these parameters with high accuracy. For example, Pishnamazi et al.^64^ and Zabihi et al.^65^ measured the decitabine and temozolomide solubility at different pressures and temperatures. After that, they correlated the measured values with maximum AARD% of 9% using semi-empirical density-based correlations which is highly acceptable for such simple correlations although one of the examined correlations namely Garlapati and Madras led to the worst ARRD% of 15.20% for temozolomide solubility.

On the other hand, further examinations revealed that using semi-empirical density-based correlations provides the chance of predicting and estimating the solubility data out of the examined ranges of temperature and pressure if the linear pattern of the used models is justified during the self-consistency test. In other words, if an acceptable level of linearity is observed for the measured solubility data, it is possible to use these models for the thermodynamic conditions that are out of the examined ranges with proper care. Among the different possible families of drugs that can be used to reduce their particle size, nonsteroidal anti-inflammatory drugs (NSAIDs) are among the most studied families due to their wide applications in relief of pain^66^.

Unfortunately, although this family is highly prescribed as a medication around the world, they have their undesired side effects. In detail, NSAIDs are a family of drugs mainly prescribed to reduce the inflammation and even pain that comes with arthritis and other musculoskeletal disorders it is undeniable to face some undesired effects including raised liver enzymes, diarrhea, headache, dizziness, salt and fluid retention, high blood pressure if these medications being used especially in the high dosage form (increase the risk of heart stroke and attack even in the people with healthy condition).

In this way, since there is no report for the solubility of lumiracoxib in the literature, the solubility of these drugs is measured in SC-CO_2_ at a temperature of 308–338 K and pressure of 120–400 bar for the first time. Lumiracoxib is a highly selective COX2 known as non-selective non-steroidal anti-inflammatory drugs (NSAIDs) which is more effective than placebo for pain relief in osteoarthritis (OA) and rheumatoid arthritis (RA), and acute pain with similar analgesic and anti-inflammatory impacts as non-selective NSAIDs and the selective COX2 inhibitor celecoxib. Besides, lumiracoxib has a lower incidence of upper gastrointestinal (GI) side effects in patients if they do not take aspirin, and a similar cardiovascular (CV) side effect similar to naproxen or ibuprofen. In this way, it seems highly effective if the size of the lumiracoxib particles is reduces for lower dosage usage for treating the diseases and relieving the pains.

Besides, the measured solubility data are modeled using four semi-empirical density based correlations namely Bartle et al., Mendez-Santiago-Teja (MST), Kumara and Johnstone (KJ), Chrastil and modified Chrastil models to find if they can accurately correlate the solubility data. Moreover, the self-consistency test is performed to know if they can extrapolate the solubility of these two drugs in SC- CO_2_ as a function of temperature and pressure.

## Experimental procedure

Model drug (nimesulide and lumiracoxib with molecular masses of 308.31 and 293.72 g/mol) was purchased from Cayman Chemical, USA with purity better than 95% (HPLC analysis). The received drugs were further purified under pressure and temperature of 450 bar and 338 K, respectively for more than 2 h to ensure the elimination of impurities existed in the drug powder since there is this possibility that impurities being dissolved in SC-CO_2_ during the experimental measurements consequently leading to undesired inaccuracies. In detail, because of using the gravimetric-based method in this work, removing the impurities enhances the accuracy of the measurements. Also, CO_2_ with a purity better than 99% was provided from Dubai Industrial Gases, Sharjah, UAE (see Table 1).

**Table 1:** Properties of the used drugs.

Name	CAS Number	Molecular Mass (g/mol)	Chemical Formula	Purity^a^ > (HPLC) (%)	Chemical Structure
Nimesulide	51,803–78-2	308.31	C_13_H_12_N_2_O_5_S	99	
lumiracoxib	220,991–20-8	293.72	C_15_H_13_ClFNO_2_	98	
Carbon dioxide	124–38-9	44	CO_2_	99	–

^a^Based on mass fraction.

### Laboratory apparatus

**Figure 1:**
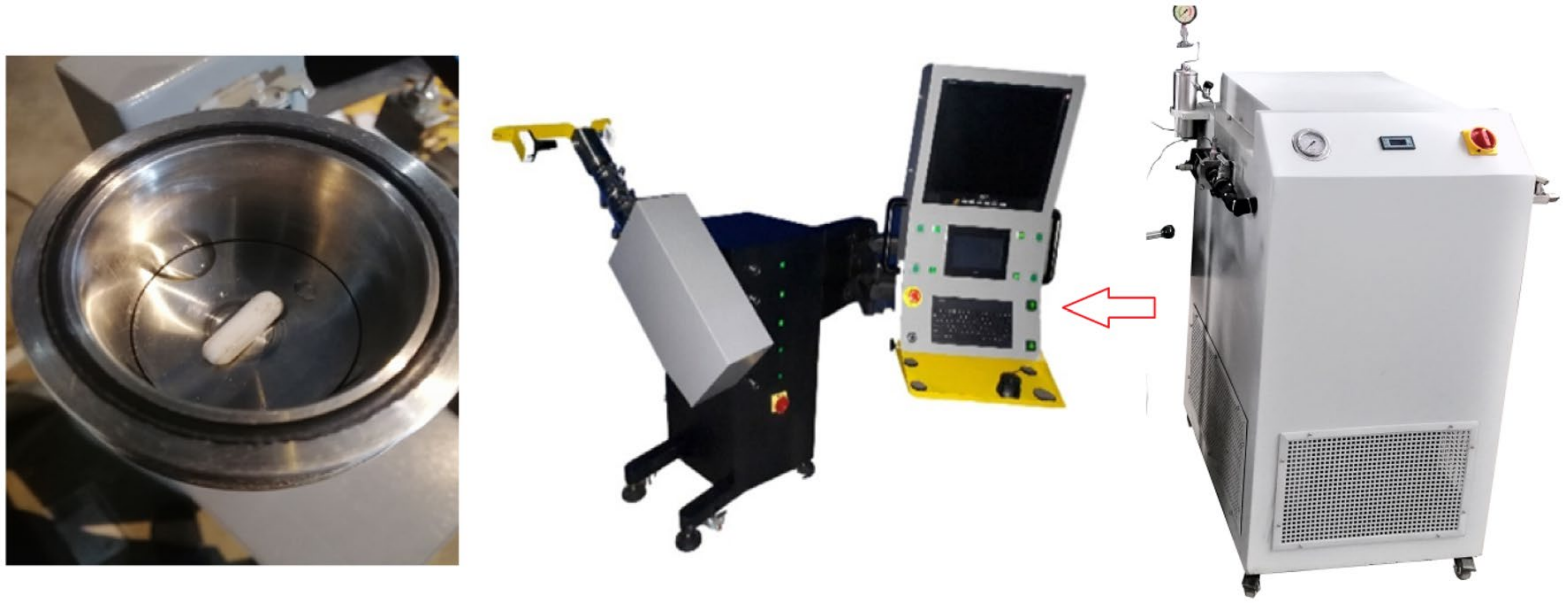
The used apparatus^14^.

A high-pressure visual chamber (volume of 400 cm^3^) (Apex Technologies Co. Arak, Iran) equipped with an internal magnetic mixer was used to measure the lumiracoxib solubility. The system also includes a high-pressure-low temperature liquefaction section (with a maximum working pressure of 200 bar and minimum temperature of − 20 °C) that allows the operator to change the gaseous CO_2_ into a liquid state before being pressurized to a desired value using an air-driven water-free non-lubricating reciprocating pump (Haskel, USA) (see Fig. 1).

As aforementioned, the used PVT cell utilizes a magnetic mixer making it possible to reach the equilibrium condition inside the main measuring chamber. The PVT cell is generally designed and rated for maximum pressures of 600 bar (Keller pressure transmitter with accuracy =  ± 35 kPa) and temperature of 426 K (with accuracy of ± 0.1 K). The used equipment is equipped with two needles and micro-metering vales for sudden depressurizing and fine controlling of the CO_2_inlet and outlet if required. The noteworthy point is that sudden depressurizing is required to prevent the deposition of drug particles in the main chamber. The point that must be mentioned is that the used PVT cell is an embedded pump-type visual PVT cell capable of the operator controlling the pressure without using the usual back pressure regulator. In other words, since the PVT cell is equipped with an automatic embedded pump, it is possible to control the pressure at a desired value with an acceptable level of accuracy without using a back pressure regulator to control the inside pressure of the main measuring chamber. In the first stage of the used procedure, the gaseous CO_2_ turns to a liquid state if it passes through a refrigeration system and is then pressurized to a desired pressure using a pneumatic high-pressure Haskel pump (USA). Before entering the pressurized CO_2_ into the main measuring chamber, the pressurized CO_2_ was delivered into a surge tank where the pressure fluctuations were dampened and the temperature was elevated.

Now the pressurized CO_2_ is ready to be transferred into the main measuring chamber which was filled with compacted drug (10 g) placed in the containment vial and glass wool. After reaching the desired pressure and temperature, the system was allowed for 3 h to reach the equilibrium while the magnetic mixer was slowly working to agitate the internal contents of the main PVT cell. This gentle agitation is required to reduce the dead volumes inside the main chamber and faster equilibrium. After 3 h, the system was suddenly depressurized and the wrapped sample was removed to be weighed. Since the weight of whole assembly (glass wool, tissue and, compacted drug) is known, it is the weight difference between the starting point and final point of the whole assembly can be considered as the dissolved amount of lumiracoxib during the measurement.

The accuracy and efficiency of the apparatus and experimental procedure were examined and validated by measuring the nimesulide solubility in a known range of temperature and different pressures as reported previously by Macnaughton et al.^48^ (see Table 2).

**Table 2:** The measured solubility of nimesulide compared with those previously measured by Macnaughton et al.^48^.

Material	Pressure	Temperature	Current work	Macnaughton et al.^48^	AARD %
nimesulide	160	312	3.22 × 10^–5^	3.18 × 10^–5^	1.24
nimesulide	190	312	5.18 × 10^–5^	5.11 × 10^–5^	1.36
nimesulide	220	312	7.53 × 10^–5^	7.42 × 10^–5^	1.48
nimesulide	160	331	3.92 × 10^–5^	3.80 × 10^–5^	3.16
nimesulide	190	331	7.19 × 10^–5^	7.08 × 10^–5^	1.55
nimesulide	220	331	9.95 × 10^–5^	9.85 × 10^–5^	1.00

At this point, it is possible to calculate the solubility of drug using the following equation (Eq. 1) and using the density of CO_2_at different pressures and temperatures reported by Fat’hi et al.^67^.

1 $${\text{y}}_{{{2 }({\text{drug}})}} = {\text{ drug}}_{{{\text{mole}}}} / \, \left( {{\text{drug}}_{{{\text{mole}}}} + {\text{ CO}}_{{{\text{2mole}}}} } \right)$$

The noteworthy point is that each reported solubility data was the average of at least three independent measurements due to the existence of uncertainties coming from the pressure and temperature fluctuations, working with high-pressure equipment, etc.

### Genetic algorithm

Over the years, different computer-based methods have been proposed and examined to simulate and model various chemical phenomena^68–71^. Among these methods utilized for prediction, estimation, and correlation, genetic algorithms (GAs) proposed by John Holland in the 60s are among the most widely used techniques as a computational analogy of adaptive systems^72^. GAs that work with selection in the presence of variation-inducing operators such as mutation and recombination (crossover) are correlated to evolution via natural selection, and using individual populations. The main principle of GAs is exactly the way that mother nature uses to survive according to the principles proposed by Charles Darwin in the first attempts.

These unique features of GA put this method on the list of methods that are flexible and capable of correlating any system with considerable complexities including engineering fields that are dealing with complicated experimentations and measurements.

In detail, using GAs not only gives this chance to solve the problem with new insight, but it consistently outperforms other traditional methods in most of the problems linked especially the problems dealing with finding optimal parameters, which might introduce several difficulties to the conventional methods. The point is that this is its high capability and performance in optimization which put the GAs in the wrong way as an optimizer which is not fair enough to consider this high potential method only as an optimizer.

In general, the GA algorithm can be described as follow:

- Random generation of initial population
- Calculating and saving the obtained results using the initial values
- Defining selection probabilities for each individual
- Generating the next set of values by probabilistically selecting individuals to produce offspring via genetic operators.
- Successive repeating of step 2 to reach the desired outcomes.

The point is that in the way of optimizing the fitting parameters, it is highly required to use some statistical parameters such as Average absolute relative deviation (AARD%), average relative deviation (ARD%), mean square error (MSE), and correlation coefficient (R2) to find the best sets of fitting parameters.

2 $$\text{MSE}=1/N{\sum }_{i}^{n}({yi}^{exp}{-{yi}^{cal.})}^{2}$$

3 $$\text{AARD\%}=1/N{\sum }_{i}^{n}\text{Abs}((({yi}^{exp}{-{yi}^{cal.})/{yi}^{exp}))100}$$

4 $${\text{R}}^{2}=\frac{\sum_{i}^{N}{{(yi}^{exp}-\stackrel{-}{y)}}^{2}-\sum_{i}^{N}{{(yi}^{exp}-{yi}^{cal.})}^{2}}{\sum_{i}^{N}{{(yi}^{exp}-\stackrel{-}{y)}}^{2}}$$

5 $$\text{ARD\%}=1/N{\sum }_{i}^{n}(({yi}^{exp}{-{yi}^{cal.})100}$$

where N, y^exp^_i_, y^cal^_i_, and y are the number of solubility data points, the i^th^ experimental value of the solubility, the i ^th^ solubility data predicted with the GA model, and the average value of the experimental solubility data. In details, to optimize the fitting parameters for any correlation or equation, a trial and error approach must be used to reach the optimum fitting parameters leading to the best MSE, AARD%, etc. values.

For this purpose, the population size was randomly varied between 20 and 100 and the stopping criteria called generation was varied between 100 and 1000, while the ranges of the three fitting parameters which must be optimized were considered between some known values at the beginning of the optimization process. After this stage, several optimized values for each fitting parameter were achieved. Then, in the next stage, the population size and generation were again changed by trial and error approach while in this point the ranges of the fitting parameters were considered between the highest and the lowest values of each fitting parameter obtained in the previous stage. This kind of procedure was performed till a desired deviation was achieved for the objective functions including MSE and AARD%.

### EoS modelling approach

In this study, the semi-empirical density-based correlations and EoS approach were used to model the measured solubility data. In this way, Esmaeilzadeh-Roshanfekr (ER) EoS along with the vdW2 mixing rule was used to measure and correlate the measured solubility data for lumiracoxib. This EoS which is developed by Esmaeilzadeh and Roshanfekr^73^ is a modified version of the cubic EoS models (Eq. 6) which its details are given elsewhere.

6 $$P= \frac{RT}{\vartheta -b}-\frac{a(T)}{\vartheta \left(\vartheta +c\right)+c(\vartheta -c)}$$

where R is the universal gas constant, “a” is a function of temperature and “b” and “c” are constants.

The used vdW2 mixing rule^74^ is as below:

7 $${a}_{m}=\sum_{i}\sum_{j}{y}_{i}{y}_{j}{a}_{ij}$$

8 $${b}_{m}=\sum_{i}\sum_{j}{y}_{i}{y}_{j}{b}_{ij}$$

9 $${a}_{ij}=\left(1-{k}_{ij}\right)\sqrt{{a}_{ii}{a}_{jj}}$$

10 $${b}_{ij}=\left(1-{l}_{ij}\right)\frac{{b}_{i}+{b}_{j}}{2}$$

11 $$\widehat{{a}_{i}}={\lceil\frac{\partial (n{a}_{m})}{\partial ({n}_{i})}\rceil}_{T, P, {n}_{i\ne j}}=2\sum_{j=1}^{N}{{a}_{ij}y}_{j}$$

12 $$\widehat{{b}_{i}}={\lceil\frac{\partial (n{b}_{m})}{\partial ({n}_{i})}\rceil}_{T, P, {n}_{i\ne j}}=2\sum_{j=1}^{N}{{b}_{ij}y}_{j}$$

The point must be mentioned is that the ER method parameters were optimized using the differential evolution (DE) method. This method is mainly based on Darwin’s theory of evolution and has been studied extensively to solve different areas of optimization and engineering applications. This method which was first proposed by Storn^75^ implements mutation, crossover, and selection as operators in its structure.

In detail, this method is a stochastic approach that simulates biological evolution. So, in the light of repeated iterations, those individuals that are adapted to the environment are preserved. However, compared with other evolutionary algorithms, DE retains the global search strategy based on population, adopts real number coding, simple mutation operation based on difference, and a one-to-one competitive survival strategy, which reduces the complexity of a genetic operation.

A close look into the optimization procedure of the DE algorithm shows that although a high similarity to the genetic algorithm (GA) approach with three main steps of mutation, crossover, and selection, the specific definitions of these operations are different from GA. In general, the DE algorithm utilizes a randomly generated initial group, uses the difference vector of two individuals randomly selected from the population as the source of the random change of the third individual, and weights the difference vector according to certain rules. After that, summing with a third individual creates a new individual which is generally called a mutation. Then, the mutant individual is mixed with a predetermined target individual to generate a test individually, and this process is called crossover. If the fitness value of the test individual is better than the fitness value of the target individual, the test individual will replace the target individual in the next generation, otherwise, the target individual will still be preserved, and this operation is called selection. In the evolution process of each generation, each vector is used as the target individual once, and the algorithm keeps good individuals and eliminates inferior individuals through continuous iterative calculation, and guides the search process to approach the global optimal solution^76^.

## Results and discussions

**Figure 2:**
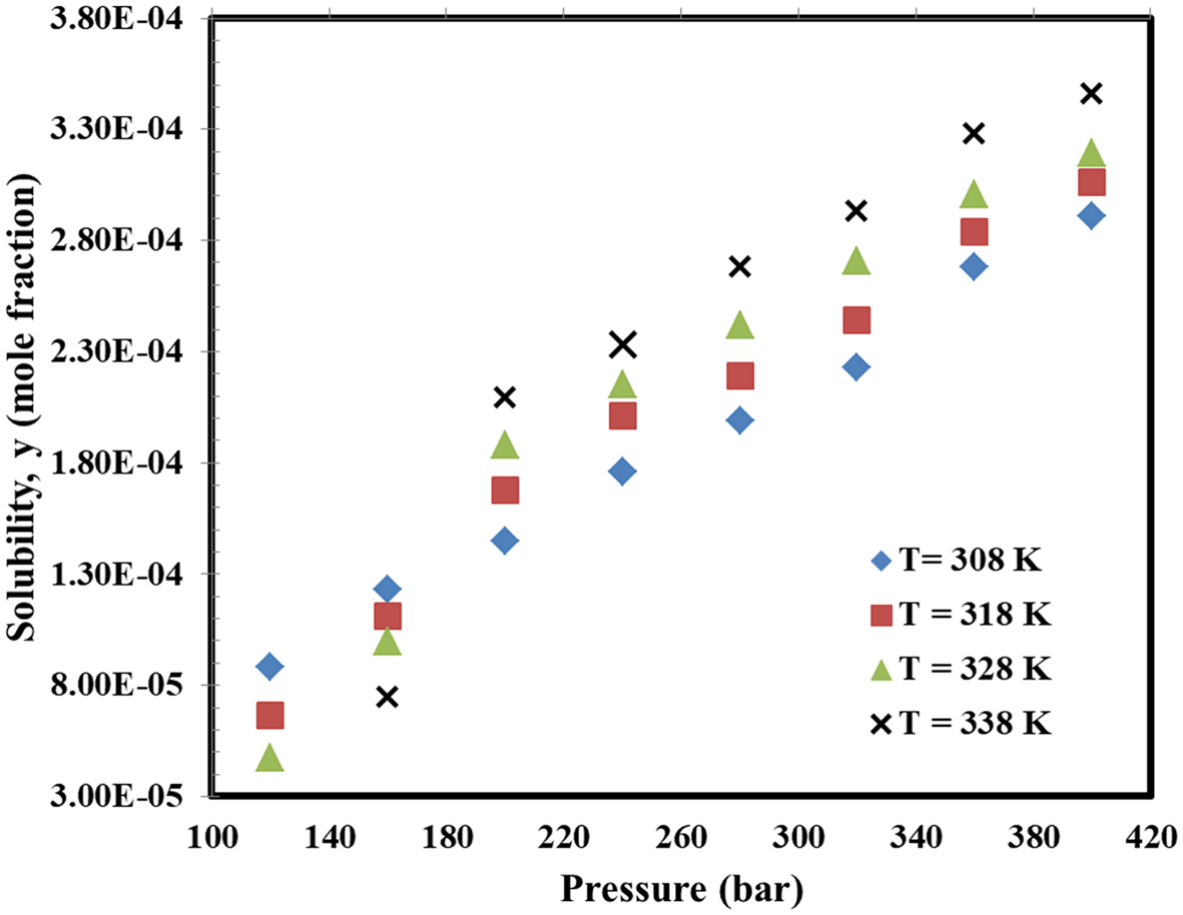
Measured solubility data along with the observed crossover pressure about 160 bar.

In the present experimental study, the main target is set to obtain the NSAID drug solubility namely lumiracoxib using a simple gravimetric-based model using variable volume PVT equipment in the pressure range of 120–400 bar and temperature range of 308–338 K. In the first stage, the used method was validated using the measurement of nimesulide as the sample drug in a specific temperature and pressure reported by Macnaughton et al.^48^. The solubility values for nimesulide showed an acceptable level of consistency for the measured solubility data although comparing the results reported by Macnaughton et al.^48^ and those measured in the current investigation revealed a systematic higher measured solubility data with AARD% of 9.16% which is an acceptable level of deviation for the used solubility measurement method. After that, the solubility of lumiracoxib was measured from 120 to 400 bar and 308 to 338 K using the validated equipment. The measured data revealed the solubility of lumiracoxib between 4.74×10^−5^ and 3.46×10^−4^ based on the mole fraction (see Fig. 2). The measured solubility data reveal that the solubility of lumiracoxib experiences a crossover pressure of about 160 bar is the accuracy of the measurements^77^. A close look into the results tabulated in Table 3 which each reported data is the average of triplicate independent measurements (with a maximum relative standard deviation percent of 8.69 % coming from pressure and temperature fluctuations and the intrinsic nature of working with high-pressure high-temperature process) revealed that as the pressure was increased between 120 and 400 bar, the solubility experienced an increase for the entire range of temperature. However, with respect to the temperature influence, the situation was a bit complex because of two competing factors namely density reduction and sublimation pressure modification. The worth mentioning point is that the main limitation of the current method is directly correlated to its principals which is weight difference. In detail, since the weight difference is directly used for calculating the solubility of the drugs, it is impossible to use this method for thermodynamic conditions with low solubility probabilities such as pressures lower than 120 bar or temperatures higher than 65 °C as the solvating power of the SC-CO_2_ reduces. The other limitation of the used method is directly correlated to the solubility of the drugs which is mostly related to the molecular weight of the drugs. In detail, the current method is not suitable to be used for the drugs with high molecular weight since they intrinsically have low solubility in SC-CO_2_ in the absence of co-solvent making it impossible to some extent to be used for solubility measurements.

**Table 3:** Solubility values of lumiracoxib.

				Temperature (K)				
Pressure (bar)	308	u (y)	318	u (y)	328	u (y)	338	u (y)
120	8.81 × 10^–5^	7.1610^–6^	6.66 × 10^–5^	4.8310^–6^	4.74 × 10^–5^	3.5910^–6^	–	–
160	1.23 × 10^–4^	9.1710^–6^	1.11 × 10^–4^	3.8610^–6^	9.97 × 10^–5^	4.6110^–6^	7.49 × 10^–5^	3.6410^–6^
200	1.45 × 10^–4^	4.2510^–6^	1.68 × 10^–4^	4.5210^–6^	1.88 × 10^–4^	1.2910^–5^	2.09 × 10^–4^	1.6710^–5^
240	1.76 × 10^–4^	7.7710^–6^	2.01 × 10^–4^	1.1910^–5^	2.15 × 10^–4^	1.3310^–5^	2.33 × 10^–4^	1.9810^–5^
280	1.99 × 10^–4^	8.1710^–6^	2.19 × 10^–4^	3.1210^–5^	2.42 × 10^–4^	8.6710^–6^	2.68 × 10^–4^	1.8910^–5^
320	2.23 × 10^–4^	1.4510^–6^	2.44 × 10^–4^	1.1110^–5^	2.71 × 10^–4^	1.1310^–5^	2.93 × 10^–4^	2.0910^–5^
360	2.68 × 10^–4^	8.4710^–6^	2.84 × 10^–4^	1.0610^–5^	3.01 × 10^–4^	7.7810^–6^	3.28 × 10^–4^	2.4910^–5^
400	2.91 × 10^–4^	2.0610^–5^	3.06 × 10^–4^	1.4310^–5^	3.19 × 10^–4^	2.1810^–5^	3.46 × 10^–4^	9.2410^–6^

Standard uncertainties, u, are u (T) = 0.1 K and u (P) = 35 kPa.

In detail, as the temperature increases, two independent variables change which can act in different ways with different effects on the substance solubility in the SC-CO_2_. In detail, increasing the temperature enhances the molecular movement and consequently causes more free movement of the molecules leading to lower density which is directly correlated to the solvating power of any solvent. In this way, as the density reduces, the solvating power experiences a reduction which means a lower amount of substance would be dissolved in the solvent. Besides, sublimation pressure modification due to temperature enhancement led to higher dissolution of drugs in the SC-CO_2_. So this is the net effect of these two factors dictates the solubility enhances or reduces and the point where this is the cumulative effect of these two competing factors changes called crossover pressure. According to these facts, it seems that there is a crossover pressure of about 160 bar for the lumiracoxib where the solubility-reducing effect of density reduction can be compensated with the sublimation pressure change causing better dissolution of substance in the SC-CO_2_.

### Solubility modelling using semi-empirical density based correlations

The lumiracoxib solubility data were correlated with density-based correlations namely Chrastil, Bartle et al., KJ, and MST models which are well-known because of their acceptable level of accuracy and simplicity needing only simple multiple linear regression (see Table 4). As it is obvious in Table 4, all of the used correlations were rather the same considering the calculated AARD% and there is no superiority between these models for the examined drugs although the Chrastil model is slightly better in an overall view. The noteworthy point is that although the level of accuracy was rather the same, each correlation has its advantages regarding the calculation of specific characteristics of the binary mixture.

**Table 4:** Fitting parameters of the used correlations for lumiracoxib.

Model (AARD %^a^)	Correlation formula	Fitting parameters		
		a	b	c
Chrastil (7.8%)	$\text{ln}s (\frac{Kg}{{m}^{3}})=a+\frac{b}{T\left(K\right)}+c.ln\rho (\frac{Kg}{{m}^{3}})$	− 3165	25.30	5.16
Mendez-Santiago-Teja (9.5%)	$\text{Tln} (\frac{y.P}{{P}^{ref}})=a+b.T \left(K\right)+c.\rho (\frac{Kg}{{m}^{3}})$	− 7957	13.776	3.062
Bartle et al. (10.7%)	$\text{Tln} (\frac{y.P}{{P}^{ref}})=a+\frac{b}{T}\left(K\right)+c.(\rho -{\rho }_{ref} )$	12.59	− 5426.5	0.009525
Kumar and Johnstone (9.8%)	$lny=a+\frac{b}{T}\left(K\right)+c.\rho (Kmol/{m}^{3})$	3.11	− 3208	0.2345
Modified Chrastil method (8.7%)	${y}_{2}= \frac{\left(\frac{{\left(\frac{RTD}{{f}^{0}}\right)}^{c-1}\text{exp}(a+\frac{b}{T})}{1+{\left(\frac{RTD}{{f}^{0}}\right)}^{c-1}\text{exp}(a+\frac{b}{T})}\right)}{1+c\left(\frac{{\left(\frac{RTD}{{f}^{0}}\right)}^{c-1}\text{exp}(a+\frac{b}{T})}{1+{\left(\frac{RTD}{{f}^{0}}\right)}^{c-1}\text{exp}(a+\frac{b}{T})}\right)}$	− 30.174	− 2000	4.61

^a^AARD % = 100 × ∑ ((y ^calc^- y ^exp^)/y ^exp^).

In detail, the first used model is Chrastil model which comprised of three fitting parameters *a*, *b* and *c* can be calculated using multiple-linear regression (see Eq. 13).

13 $$\text{ln}s (\frac{Kg}{{m}^{3}})=a+\frac{b}{T\left(K\right)}+c.ln\rho (\frac{Kg}{{m}^{3}})$$

**Figure 3:**
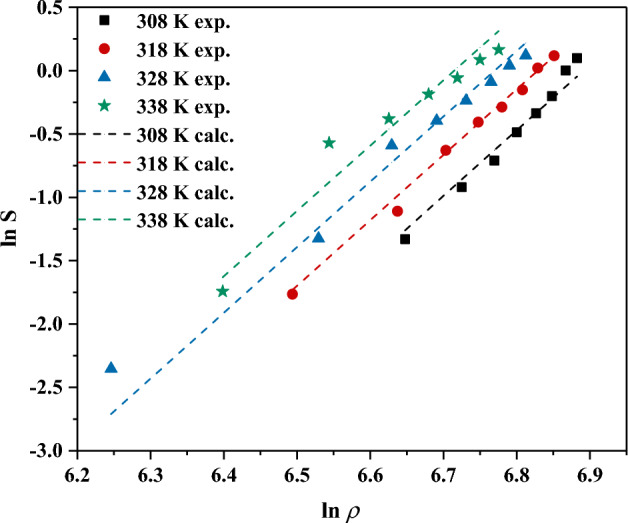
The measured solubility data and the correlated ones using Chrastil model.

On the other side, there are known and specific parameters including *s* and *ρ* which are the solubility and the density of CO_2_ at the experimental absolute *T* and *p*. The point is that the theoretical background of this correlation is based on a well-known theory called association theory. This theory deals with the concept that each solute molecule is surrounded by *c* molecules of solvent in a mixture. According to its basics and concepts, it is possible to estimate two important parameters namely the enthalpies of vaporization and solvation using the fitting parameters of *a* which is equal to ΔH_total_/R, where ΔH_total_ is the sum of enthalpies of vaporization and solvation of the solute and R is the universal ideal gas constant. According to this information, the measured solubility data and performed modeling using the Chrastil model revealed that the total and solvation enthalpies for lumiracoxib are 26.31 and 210.3 kJ/mol, respectively (see Fig. 3).

In the next phase of this section, the modified Chrastil method^78^ was used to find the potential of the Chrastil model compared with the modified Chrastil model. In contrast to the other used correlations in the current investigation which required a multiple linear regression approach to obtain the fitting parameters, the modified Chrastil model fitting parameters were r calculated and optimized using the genetic algorithm approach previously described and discussed.

14 $${y}_{2}= \frac{\left(\frac{{\left(\frac{RTD}{{f}^{0}}\right)}^{c-1}\text{exp}(a+\frac{b}{T})}{1+{\left(\frac{RTD}{{f}^{0}}\right)}^{c-1}\text{exp}(a+\frac{b}{T})}\right)}{1+c\left(\frac{{\left(\frac{RTD}{{f}^{0}}\right)}^{c-1}\text{exp}(a+\frac{b}{T})}{1+{\left(\frac{RTD}{{f}^{0}}\right)}^{c-1}\text{exp}(a+\frac{b}{T})}\right)}$$

where f is a known reference state and it may be chosen as unity or critical pressure of the SF or any other known value, R is the universal gas constant, T is the temperature in Kelvin, D is the density, y2 is the solubility, and the c, a, and b are the fitting parameters must be optimized using the optimization approaches which the genetic algorithm was used in the current investigation. Using the optimized fitting parameters which are c = 4.61, a = − 30.174, and b = − 2000 leading to solubility calculation with AARD% of 8.7% which is slightly higher than the Chrastil model 7.8%. In other words, there is no superiority between the modified Chrastil model and the Chrastil model for the lumiracoxib solubility measured in the current investigation. In other words, although calculating the fitting parameters of the modified Chrastil model required a more complicated optimization procedure than the Chrastil model only required two linear regressions, the method was not capable of providing better AARD% during the solubility prediction stage.

The next examined correlation is the one proposed by Méndez-Santiago^60^ in 1999 and is among the most widely used and accurate correlations using only three fitting parameters. This model is basically based on a model that needs sublimation pressure which is impossible in most cases to measure experimentally. So, the general form was modified with the assistance of Clausius − Clapeyron expression to the following form which requires no sublimation pressure information.

15 $$\text{Tln} (\frac{y.P}{{P}^{ref}})=a+b.T \left(K\right)+c.\rho (\frac{Kg}{{m}^{3}})$$

**Figure 4:**
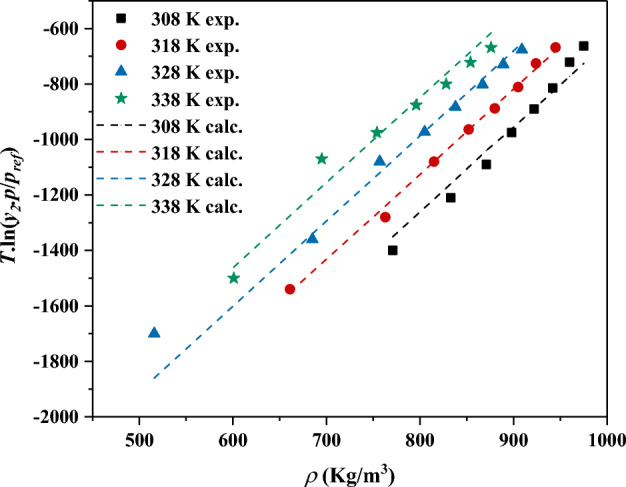
The correlated solubility data using MST model.

In the above equation, *p*^*ref*^ refers to the standard pressure of 0.1 MPa, *y* refers to the solubility and *a*, *b*, and *c* are the fitting parameters cab neb calculated using multiple linear regressions (see Fig. 4).

**Figure 5:**
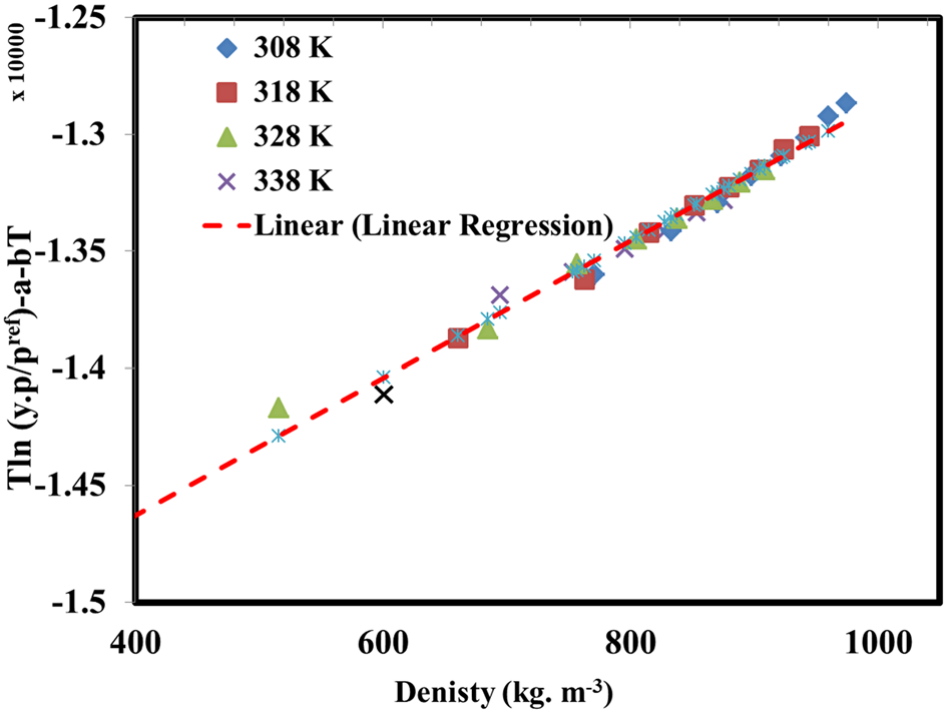
The performed self-consistency test using MST model.

The worth mentioning point is that the performed self-consistency test using MST mode (see Fig. 5) revealed the correlative and extrapolative capabilities of the MST model through the 160–400 bar and 308–338 K for pressure and temperature, respectively, or out of these examined ranges. The reason behind this claim is that the performed self-consistency revealed that the measured solubility data form a linear pattern during the self-consistency test even for densities out of the examined range which means the extrapolative capability of the examined correlation.

**Figure 6:**
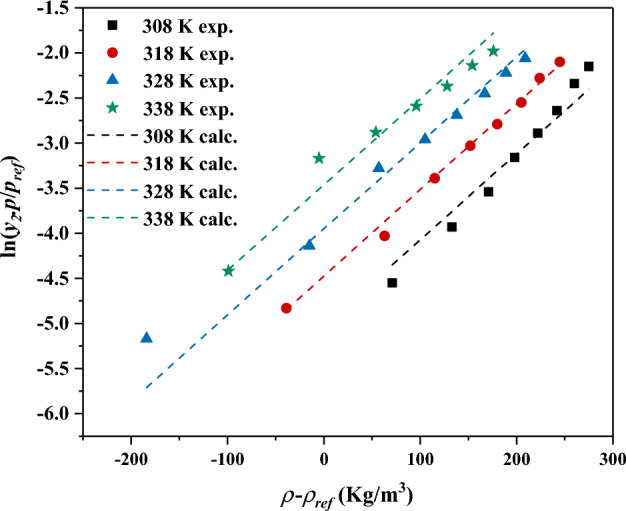
The calculated solubility data of lumiracoxib using Bartle et al. model.

The Bartle et al.^63^ model was the third examined correlative model used to predict/correlate the solubility of lumiracoxib in SC-CO_2_ in wide ranges of temperature and pressure similar to the previously used correlations has three fitting parameters must be calculated using multiple linear regression approach (see Eq. 16 and Fig. 6).

16 $$\text{Tln} (\frac{y.P}{{P}^{ref}})=a+\frac{b}{T}\left(K\right)+c.(\rho -{\rho }_{ref} )$$

In the above equation, *y*, *p*^*ref*^ , *p, ρ, and ρ*_*ref*_ refer to the solubility of lumiracoxib based on the mole fraction, the reference pressure of 0.1 MPa, the CO_2_ density, the reference density of 700 kg m^−3^^63^.

One of the most important advantages of the current method is its lower sensitivity to the density variation as a function of pressure and temperature using *ρ*_*ref*_. Similar to the other examined correlations, it is required to use multiple linear regression steps to obtain the fitting parameters. In detail, for the first stage, the ln(y·p/P_ref_) values must be depicted vs density to find a straight line. At this point, this is the slope of the straight line must be used for the next stage of regression. In the ideal cases, the slope of all the examined isotherms must be the same. But, in the real situation, different slopes would be calculated for each isotherm. Respecting this fact, it is the average of the slopes of different lines (for different isotherms) must be used for the next regression step. So, after calculating the average of four slopes, *a* and *b* parameters can be obtained using the last linear data regression. Similar to the Chrastil model, the Bartle et al.^63^ model can be used to estimate the vaporization enthalpy, ΔH_total_, as follows: -R*b* and then it the heat of solvation can be calculated based on Hess’s law.

The last used correlations is KJ^59^ (Eq. 10) model which can be utilized to correlate the solubility of substance using density of SC-CO_2_.

17 $$lny=a+\frac{b}{T}\left(K\right)+c.\rho (Kmol/{m}^{3})$$

where *y* refers to the solubility of solute in SC-CO_2_, *T* refers to temperature, ρ refers to the density of supercritical CO_2_ at specific pressure and temperature while *a*, *b* and *c* are the fitting parameters can be calculated using multiple regression.

In the last stage of this investigation, the measured solubility data were compared with those reported in different literature to find if there is any correlation between the physiochemical properties of the drugs and their solubility in SC- CO_2_. In this way, the solubility of 9 drugs was tabulated in Table 5 and compared with the solubility data obtained for lumiracoxib in the pressure and temperature ranges of 120–400 bar and 308–338 K, respectively. At first glance, there is a direct relation between the drug solubility and the molecular weight. In detail, some of the measured solubility data revealed that as the molecular weight increases, the solubility of the drug in SC- CO_2_reduces by several orders. However, further investigation revealed that this trend is not a generalized relation for the molecular weight and solubility data. For example, the solubility for amiodarone hydrochloride with a molecular weight of 681.77 is higher than the solubility data measured for esomeprazole with a molecular weight of 345.42 g gmol^−1^. On the other sides, further examinations showed that the other effective parameter on the solubility of the drug is its structure and the number of rings that existed in its structure. In detail, the results revealed that as the number of the rings increases and the number of branches in the molecular structure reduces; the solubility of the drug reduces as is highly obvious for imatinib mesylate with a molecular weight of 589.71 g gmol^−1^ with the highest number of rings and the zero number of branches leading to the lowest solubility data of 4.8610^−7^ to 0.4.0610^−6^ based on the mole fraction. However, amiodarone hydrochloride with a molecular weight higher than imatinib mesylate but with branches in its structure provides better solubilization in the SC- CO_2_in the range of 2.510 × 10^−5^ to 1.012 × 10^−3^ based on the mole fraction.

**Table 5:** The physiochemical properties and solubility of different drugs.

Drug	Mw (g·mol^−1^)	Molecular Formula	Molecular structure	Pressure (bar)	Temperature (K)	Solubility (mole fraction)	Ref
Lumiracoxib	293.72	C_15_H_13_ClFNO_2_		120–400	308–338	4.74 × 10^–5^-3.46 × 10^–4^	Current investigation
Triamcinolone Acetonide	434.5	C_24_H_31_FO_6_		120–270	308–338	6.90 × 10^−6^ to 2.13 × 10^−4^	^79^
Imatinib mesylate	589.71	C30H35N7O4S		120–270	308–338	4.8610^–7^ to .4.0610^–6^	^32^
Aprepitant	534.435	C_23_H_21_F_7_N_4_O_3_		120–330	308–338	0.45 × 10^−5^–7.67 × 10^−5^	^80^
Amiodarone hydrochloride	681.77	C_25_H_30_ClI_2_NO_3_		120–270	308–343	2.510 × 10^−5^ to 1.012 × 10^−3^	^81^
Loratadine	382.89	C_22_H_23_ClN_2_O_2_		120–270	308–338	0.45 × 10^−5^ to 1.3016 × 10^−3^	^82^
Ketotifen fumarate	425.5	C_23_H_23_NO_5_S		120–300	308–338	0.211 × 10^−4^ to 10.766 × 10^−4^	^83^
Esomeprazole	345.42	C_17_H_19_N_3_O_3_S		120–270	308–338	1.11 × 10^−5^ to 9.10 × 10^−4^	^84^
Azathioprine	277.26	C_9_H_7_N_7_O_2_S		120–270	308–338	0.27 × 10^−5^ to 1.83 × 10^−5^	^85^
Lansoprazole	369.36	C_16_H_14_F_3_N_3_O_2_S		120–270	308–338	1.15 × 10^−5^ to 7.36 × 10^−4^	^86^
Sunitinib malate	532.5612	C_26_H_33_FN_4_O_7_		120–270	308–338	0.5 × 10^−5^ to 8.56 × 10^−5^	^87^

According to these findings, it can be concluded that the simple gravimetric method for solubility measurement is not a good candidate for the solids and drugs with the low branches and high rings in its structure due to a very low solubility makes it impossible to measure their solubilities using gravimetric methods. Besides, it can be concluded that using SC-CO_2_-based technologies for micronizing and reducing the size of the drugs with structures with no branched sections in their structure and a high number of rings is inefficient due to probable low solubility limits even under high pressure of 300 bar which can be considered as one of the limitations of using SC- CO_2_ for drug particle size and morphology modifications.

### EoS approach for solubility modelling

In the last stage of this investigation, the Esmaeilzadeh-Roshanfekr (ER) EoS approach was used to model the measured solubility data (see Table 6). For this purpose, it was necessary to predict the physio-chemical properties of the drug including critical temperature, critical pressure, and acentric factor using the Joback^88^ and Constantinou and Gani group contribution methods^89^.

**Table 6:** The results of solubility modelling using EoS (Tc = 768.76 K, Pc = 18.18 bar using Joback method^88^, and acentric factor = 0.9875 using Constantinou and Gani^89^ along with Vs = 215 cm^3^/mol).

EoS	ER EoS (using DE optimization approach)		
T (K)	Kij	Lij	AARD
308.15	0.2248	0.2125	7.86
318.15	0.1201	0.0819	8.90
328.15	0.0022	-0.0678	14.28
338.15	-0.1299	-0.2403	10.28

The obtained results revealed that the used EoS was capable of correlating the solubility data with the assistance of the DE approach for optimization of the coefficient with minimum and maximum AARD of 7.86 and 14.28%, respectively. A glance into the results revealed that the used EoS method is as accurate as the used semi-empirical density-based correlations for temperatures of 308.15, 318.15, and 338.15 K, and the highest AARD% was obtained for temperatures of 328.15 K. The point is that the obtained deviations for EoS not only can be correlated to the fluctuations of working with high temperatures and pressures that appear during the measurement stage, but also the other source of deviations in the capability of the used ER method can be correlated to the using the estimated Tc, Pc, and acentric factor since the experimental values are not available. In other words, the accuracy of the EoS method including ER EoS can be improved if the experimental values of Tc, Pc, and ω were inserted into the EoS modeling approaches which are not unfortunately available at the current time.

To sum up, considering the obtained results using two approaches of semi-empirical density-based correlations and EoS, it can be concluded that the semi-empirical density-based correlations are more applicable since they use parameters that their experimental values exist and easily can be measured. However, the EoS approach not only uses estimated necessary physio-chemical properties, but it is also a complex method that requires some special optimization strategies to find the fitting parameters.

## Conclusions

The present experimental work is concentrated on the measuring the solubility of lumiracoxib in SC-CO_2_ in temperatures of 308 to 338K, and pressures of 120 to 400 bar. The solubility data were measured based on a gravimetric method as the main core of the procedure coupled with the static method of solubility measurement using a variable volume PVT cell and gas-booster unit to maintain the desired pressure. In this way, before measuring the solubility of lumiracoxib, the solubility of nimesulide was measured using the proposed gravimetric-based method to ensure about the accuracy and validity of the used experimental procedure and apparatus, respectively.

The solubility data which were between 4.74 × 10^−5^ and 3.46 × 10^−4^ based on the mole fraction revealed that the pressure has a direct influence on the enhancement of solubility while the temperature effect is more complex than pressure. In detail, the measurements revealed a changing pressure point called crossover pressure around 160 bar which the effect of temperature turns to an increasing effect while for the pressures below this crossover point the temperature effect is decreasing point. The observed trend was correlated to the interactions between the density reduction and sublimation pressure modification and the cumulative effect of these two factors. After that, the measured solubility data were correlated using density-based correlations all of them use three adjustable parameters which can be calculated with the assistance of the multiple linear regression method. The performed regression approach and calculation the fitting parameters revealed that using those fitting parameters capable the operator to correlate the solubility data with AARD% of 10.7%, 9.5%, 9.8%, 7.8%, and 8.7% for Bartle et al., Mendez-Santiago-Teja (MST), Kumar and Johnstone (KJ), Chrastil, and modified Chrastil models, respectively which are an acceptable level of accuracy. Moreover, according to the self-consistency test performed for the MST model, it can be concluded that not only it is possible to correlate the solubility of lumiracoxib using these semi-empirical correlations but also it is possible to extrapolate the drug solubility for the temperatures and pressures beyond the examined range in the current investigation which is undeniably a significant capability for the examined models. To sum up, it seems that based on the measured solubility data, using SC-CO_2_-based particle formation technologies is an acceptable approach for producing micron or nano-size particles of lumiracoxib for better efficiency of this drug.

## Data Availability

All data generated or analyzed during this study are included in this published article.

## Abbreviations

a: Fitting parameter
AARD%: Average absolute relative deviation percent
ARD%: Average relative deviation percent
b: Fitting parameter
c: Fitting parameter
CV: Cardiovascular
D: Density
EoS: Equations of state
GA: Genetic algorithm
GI: Gastrointestinal
GAS: Gas anti-solvent
KJ: Kumar and Johnstone
MSE: Mean square error
MST: Mendez-Santiago-Teja
N: Number of solubility data
NSAIDs: Nonsteroidal anti-inflammatory drugs
OA: Osteoarthritis
P: Pressure
P^ref^: Reference pressure, 0.1 MPa
R: Universal gas constant
RA: Rheumatoid arthritis
R2: Correlation coefficient
s: Solubility, Kg m^−3^
SC-CO_2_: Supercritical carbon dioxide
SFT: Supercritical fluid technology
SAS: Supercritical anti-solvent
T: Temperature
y^exp^_i_: The ith experimental value of the solubility
y^cal^_i_: The ith solubility data predicted
ρ: Density, Kg M^−3^ or Kmol m^−3^
ΔH_total_: Sum of enthalpies of vaporization and solvation
ρ_ref_: Reference density equal 700 kg m^−3^
